# Evaluation of the anti-diarrheal activity of the leaf extract of *Croton macrostachyus* Hocsht. ex Del. (Euphorbiaceae) in mice model

**DOI:** 10.1186/s12906-016-1357-9

**Published:** 2016-09-29

**Authors:** Amsalu Degu, Ephrem Engidawork, Workineh Shibeshi

**Affiliations:** 1 Department of Pharmacy, College of Medicine and Health Sciences, Ambo University, P.O, Box 19, Ambo, Ethiopia; 2 Department of Pharmacology and Clinical Pharmacy, College of Health Sciences, School of Pharmacy, Addis Ababa University, P.O. Box 1176, Addis Ababa, Ethiopia

**Keywords:** Antidiarrheal activity, Castor oil induced diarrhea, Gastrointestinal transit, Anti-enteropooling, *Croton macrostachyus*

## Abstract

**Background:**

Traditional healers in Ethiopia use a wide range of medicinal plants with antidiarrheal properties. Among these, *Croton macrostachyus* is one such plant claimed to have an antidiarrheal activity in Ethiopian folklore medicine. Previous studies showed that the crude extract is endowed with the claimed property. The present study was undertaken to further the claim by screening different fractions for the said activity so that it could serve as a basis for subsequent studies.

**Methods:**

The fractions were obtained by successive extraction in soxhlet apparatus with solvents of different polarity (chloroform & methanol) followed by cold maceration of the deposit of the methanol fraction with distilled water. The antidiarrheal activity was evaluated using castor oil induced diarrheal model, charcoal meal test and anti-enteropooling test in mice. The test groups received various doses (300, 400, 500 mg/kg and an additional dose of 1000 mg/kg for the aqueous fraction) of the fractions, whereas positive controls received either Loperamide (3 mg/kg) or Atropine (5 mg/kg) and negative controls received vehicle (10 ml/kg).

**Results:**

In the castor oil induced model, the chloroform (at all test doses) and methanol (at 400 & 500 mg/kg) fractions significantly delayed diarrheal onset, decreased stool frequency and weight of feces. The aqueous fraction was however devoid of significant effect at all the tested doses. Chloroform and methanol fractions produced a significant dose dependent decline in the weight and volume of intestinal contents while the aqueous fraction did not have a significant effect. All the fractions produced a significant anti-motility effect either at all doses (chloroform fraction) or at middle and higher doses (methanol and aqueous fractions).

**Conclusion:**

The present study demonstrated that the chloroform and methanol fractions possessed significant anti-diarrheal activity. Nevertheless, the aqueous fraction showed only significant anti-motility effect at the higher dose (1000 mg/kg) employed in the study.

## Background

Diarrheal diseases account for one in nine child deaths worldwide, making diarrhea the second leading cause of death among children under the age of five and responsible for killing around 760, 000 children every year [1, 2]. The global burden of diarrheal incidence and severity of the disease is highest in Southeast Asian and African regions [3], with the highest childhood deaths being reported in Sub-saharan Africa [4]. In Ethiopia, diarrhea disease is a major public healthconcern as it results in high childhood mortality and mortality [5–8].

Herbal medicines cater about 80 % of the health needs of world’s population, especially for millions of people in the vast rural areas of developing countries [9, 10]. In Ethiopia, a wide range of medicinal plants have been widely used for the management of diarrhea without scientific investigation of its safety and therapeutic potentials [11, 12].

Among these, *Croton macrostachyus* Hochst.ex Del. (Euphorbiaceae) which is commonly known as broad-leaved *Croton* (English), Bisana (Amharic), Tambuk and Tambush (Tigrigna), and Abnga in Berta ethnic group [13, 14] is endowed with a number of ethnomedicinal uses in Ethiopia [12, 13, 15, 16]. Ethnopharmacological studies revealed that hydroalcoholic extracts of *C.macrostachyus* leaves have promising activity against *Neisseria gonorrhoeae* [17], *Plasmodium berghei* [18] and *Mycobacterium tuberculosis* [19]. It also has analgesic and anti-inflammatory [20], anti-convulsant and sedative [21] and anti-leishmanial activities [22].

In addition, it has previously been demonstrated that the crude leaves extract of C. *macrostachyus* has remarkable antidiarrhoeal activity in an animal model of diarrhea [41]. However, it is not known which solvent fractions possess antidiarrheal activity. The present study aims to investigate the antidiarrheal activity of the solvent fractions of *C.macrostachyus* leaves in mice.

## Methods

### Drugs and chemicals

Castor oil (Amman Pharmaceutical Industries, Jordan), activated charcoal (Acuro Organics Ltd, New Delhi), Loperamide (Daehwa Pharmaceuticals, Republic of Korea), Atropine sulphate (Lab Renaudin, France), distilled water (Ethiopian Pharmaceutical Manufacturing Factory, Ethiopia), Tween 80 (Atlas Chemical Industries Inc, India), chloroform (Finkem Laboratory Reagent, India), and methanol (Fisher Scientific, UK) were used in the study.

### Plant material

Leaves of *C.macrostachyus* were collected from Kolfe keranio subcity of Addis Ababa City Administration in October 2013. The plant was authenticated and a voucher specimen (number AD001) was deposited at the National Herbarium of Addis Ababa University for future reference. The leaves were washed using distilled water, dried at room temperature under shade for 14 days and then pulverized to coarse powder using mortar and pestle.

### Experimental animals

Healthy Swiss albino mice of either sex, weighing 20–30 g and aged 6–8 weeks were used for the experiment. The animals were obtained from Ethiopian Public Health Institute and School of Pharmacy, Addis Ababa University and kept in plastic cages at room temperature and on a 12 h light–dark cycle with free access to pellet food and water ad libitum. The animals were acclimatized to laboratory condition for 1 week prior to the experiments [23]. All studies were conducted in accordance with the guideline for the care and use of laboratory animals [24].

### Solvent fractionation

Two hundred gm dry powder of the plant material was subjected to successive soxhlet extraction with solvents of different polarity (chloroform and methanol) followed by cold maceration with water. First, 50 mg of the powdered plant material was placed in the extraction chamber of the Soxhlet apparatus. The extracting solvent (chloroform) in the flask was heated until clear liquid contents of the chamber siphoned into the solvent flask. Each time 50 mg of the powdered plant material was extracted with 200 ml of solvent in the soxhlet extraction process [25]. The chloroform fraction was then filtered with Whatman No. 1 filter paper and concentrated using rotary evaporator (Buchi labortechnik AG, Switzerland) under reduced pressure set at 40 °C followed by oven at room temperature for 12 h [26]. The residue was collected and dried at room temperature to remove chloroform.

The plant material was then dried and extracted using methanol following the same procedure as described before to get the methanol fraction. Finally, the residue of methanol fraction was collected and dried at room temperature. Then, the whole dried residue was cold macerated in an Erlenmeyer flask with distilled water and allowed to stand at room temperature for a period of 72 h with occasional shaking using mini orbital shaker (Bibby scientific limited stone Staffordshire, SI150SA, UK). It was then filtered two times with gauze then through whatman filter paper (NO.1). The residue was re- macerated two times for a total of 6 days in order to obtain a better yield. The marc was pressed, and the combined liquid was clarified by filtration and then the filtrate was freeze dried in a lyophilizer (Operan, Korea vacuum limited, Korea) to remove water. After drying, percentage yield of all fractions were determined and the yield was of 4.7, 5.6, and 3.5 % for the chloroform, methanol and aqueous fractions, respectively. The chloroform and methanol fractions were reconstituted in 2 % Tween- 80, while the aqueous fraction was reconstituted in distilled water.

### Grouping and dosing

The study was conducted using 30 mice for each fraction. The mice were randomly assigned into three treatment groups and two controls, six mice per group for each fraction. Negative controls were treated with the vehicle used for reconstitution (2 % v/v Tween 80 for the chloroform and methanol fractions or distilled water for the aqueous fraction) orally using oral gavage. The second group was assigned as positive control and treated with standard drugs (3 mg/kg Loperamide orally for anti-enteropooling test & castor oil induced diarrhea, 5 mg/kg Atropine sulphate intraperitoneally for charcoal meal test). Treatment groups were treated with various doses of the fractions (300, 400, 500 mg/kg for the chloroform and methanol fractions, and an additional dose of 1000 mg/kg for the aqueous fraction). Doses were selected based on acute toxicity studies. The fractions were reconstituted with the respective vehicles during the day of experiment and administered orally using oral gavage.

### Determination of antidiarrheal activity

#### Castor oil induced Diarrhea

Swiss albino mice of either sex were fasted for 18 h with free access to water and randomly allocated and treated as described under grouping and dosing. One hour after administration, all animals were given 0.5 ml of castor oil orally and individually placed in cages in which the floor was lined with transparent paper and changed every hour. During an observation period of 4 h, the time of onset of diarrhea, frequency of defecation and weight of feces excreted by the animals was recorded [23]. The percentage of diarrheal inhibition was determined according to the following formula [27, 28].

$$ \%\;\mathrm{of}\;\mathrm{inhibition}=\frac{\mathrm{Average}\;\mathrm{number}\;\mathrm{of}\;\mathrm{W}\mathrm{F}\mathrm{C}-\mathrm{Average}\;\mathrm{number}\;\mathrm{of}\;\mathrm{W}\mathrm{F}\mathrm{T}}{\mathrm{Average}\;\mathrm{number}\;\mathrm{of}\;\mathrm{W}\mathrm{F}\mathrm{C}}*100 $$

Where, WFC = average number of wet feces in control group and WFT = average number of wet feces in test group.

Calculations were made for the delay in diarrhoeal onset and purging index by comparing with the control group. The in vivo anti-diarhhoeal index (ADI) was then expressed according to the formula [29] described below.

$$ In\; vivo\; anti\; diarrheal\; index\;(ADI)=\sqrt[3]{Dfreq\times Gmeq\times Pfreq} $$

Where: Dfreq = Delay in defecation time or diarrheal onset (in % of control), Gmeq = Gut meal travel reduction (in % of control) and Pfreq = purging frequency as number of wet stool reduction (in % of control).

### Charcoal meal (gastrointestinal motility) test

Mice of either sex were fasted for 18 h with free access to water and divided and treated according to their respective groupings 1 h before oral administration of 0.5 ml castor oil. 1 ml of a marker (5 % charcoal suspension in 2 % Tween 80) was administered orally 1 h after castor oil treatment. The animals were then sacrificed 1 h after administration of the marker and the small intestine was dissected out from pylorus to caecum. The distance travelled by charcoal meal from the pylorus was measured [30]. Then, the percentage of inhibition and Peristalsis index was expressed using the following formula [28, 29, 31].

$$ \mathrm{Percentage}\;\mathrm{of}\;\mathrm{inhibition}=\frac{\mathrm{A}-\mathrm{B}}{\mathrm{A}}*100 $$

where A is the distance (cm) moved by the charcoal in the control group, and B is the distance(cm) moved by the charcoal in the treated group.

$$ \mathrm{Peristalsis}\;\mathrm{index}=\frac{\mathrm{mean}\;\mathrm{distance}\;\mathrm{traveled}\;\mathrm{b}\mathrm{y}\;\mathrm{charcoal}\;\mathrm{meal}}{\mathrm{mean}\;\mathrm{length}\kern0.5em \mathrm{of}\ \mathrm{small}\;\mathrm{intestine}}*100 $$

### Anti-enteropooling test

Intraluminal fluid accumulation was determined using the method described by Islam et al. [32]. Animals of either sex were deprived both food and water for 18 h, and grouped and treated as described under grouping dosing. After 1 h, 0.5 ml of castor oil was administered orally. 1 h later, the mice were sacrificed by cervical dislocation and the small intestine was then dissected out and weighed. After which, intestinal contents were collected by milking into a graduated tube and the volume was measured. The intestine was reweighed and the difference between the full and the empty intestine was calculated [32]. Finally, percentage of reduction of intestinal secretion and weight of intestinal contents was determined using the following formula [28].

$$ \mathrm{Mean}\;\mathrm{Percentage}\;\mathrm{inhibition}=\frac{\mathrm{MVICC}-\mathrm{MVICT}}{\mathrm{MVICC}}*100 $$

Where, MVICC is the mean volume of the intestinal content of the control group while MVICT is the mean volume of the intestinal content of the test group.

$$ \mathrm{Mean}\;\mathrm{percentage}\;\mathrm{inhibition}\;\mathrm{of}\;\mathrm{intestinal}\;\mathrm{content}\;\mathrm{weight}=\frac{A-B}{A}*100 $$

Where A is the mean weight of intestinal content of the control and B is the mean weight of intestine content of the test group.

### Preliminary phytochemical screening

The qualitative phytochemical investigations of the chloroform, absolute methanol and aqueous fractions of *C.macrostachyus* leaves were carried out using standard tests like Mayer’s test for alkaloids, Liberman –Burchard test for steroids, Salkowski test for terpenoids, Keller-Kilani test for glycosides and Ferric chloride test for tannins [33].

### Test for terpenoids (Salkowski test)

To 0.5 g of each solvent fraction of *C.macrostachyus* leaves, 2 ml of chloroform was added. Then, 3 ml concentrated sulfuric acid was carefully added to form a layer. A reddish brown coloration of the interface indicates the presence of terpenoids.

### Test for Saponins

To 0.5 g of each fraction, 5 ml of distilled water was added in a test tube. Then, the solution was shaken vigorously and observed for a stable persistent froth. Formation of froth indicates the presence of Saponins.

### Test for tannins

About 0.5 g of each fraction was boiled in 10 ml of water in a test tube and then filtered. A few drops of 0.1 % ferric chloride were added. A brownish green or a blue-black precipitate indicated the presence of tannins.

### Test for flavonoids

About 10 ml of ethyl acetate was added to 0.2 g of each fraction and heated on water bath for 3 min. The mixture was cooled and filtered. Then, About 4 ml of the filtrate was shaken with 1 ml of dilute ammonia solution. The layers were allowed to separate and the yellow color in the ammoniacal layer indicated the presence of Flavonoids.

### Test for cardiac glycosides (Keller-Killiani test)

To 0.5 g of each fraction diluted to 5 ml in water was added 2 ml of glacial acetic acid containing one drop of ferric chloride solution. This was underlayed with 1 ml of concentrated Sulfuric acid. A brown ring at the interface indicated the presence of a deoxysugar characteristic of cardenolides. A violet ring may appear below the brown ring, while in the acetic acid layer a greenish ring may form just above the brown ring and gradually spread throughout this layer.

### Test for steroids

Two ml of acetic anhydride was added to 0.5 g fraction of each sample with 2 ml sulfuric acid. The color changed from violet to blue or green in some samples indicating the presence of steroids.

### Test for alkaloids

0.5 g of extract was diluted to 10 ml with acid alcohol, boiled, and filtered. To 5 ml of the filtrate, 2 ml of dilute ammonia and 5 ml of chloroform was added and shaken gently to extract the alkaloidal base. The chloroform layer was extracted with 10 ml of acetic acid. This was divided into two portions. Mayer’s reagent was added to one portion and Draggendorff’s reagent to the other. The formation of a cream (with Mayer’s reagent) or reddish brown precipitate (with Draggendorff’s reagent) was regarded as positive for the presence of alkaloids.

### Oral acute toxicity

Two groups of six female Swiss albino mice were used for each fraction. After being fasted for 2 h, mice in the first group were given 2 g /kg and the second group 5 g/kg of each fraction orally and observed for any signs of toxicity daily for 14 days to assess safety of the extract. Animals were observed for gross changes such as loss of appetite, hair erection, lacrimation, tremors, convulsions, salivation, diarrhoea, mortality and other signs of overt toxicity [34].

### Statistical analysis

Data are expressed as mean ± standard error of the mean (SEM) and analyzed using the Statistical Package for the Social Sciences (SPSS), version 16.0 software. Difference between group means was analyzed with one way analysis of variance (ANOVA) followed by Tukey post Hoc test. *P* <0.05 was considered as statistically significant.

## Results

### Oral acute toxicity study

The acute toxicity study indicated that the fraction caused no mortality in both doses (2 and 5 g/kg) within the first 24 h as well as for the following 14 days. Physical and behavioral observations of the experimental mice also revealed no visible signs of overt toxicity like lacrimation, loss of appetite, tremors, hair erection, salivation, diarrhea and the like. This suggests that LD50 of the extract is greater than 5 g/kg.

### Effects on castor oil- induced diarrhea in mice

In the castor oil-induced diarrheal model (Table 1), the chloroform (at all doses tested) and methanol fraction (at 400 & 500 mg/kg) of *C. macrostachyus* leaves significantly delayed the time of diarrheal onset and stool frequency in a dose-dependent manner. In addition, at all the tested doses, the chloroform fraction produced comparable effect with the standard drug, Loperamide (81.5 %). On the contrary, the aqueous fraction was devoid of significant anti-diarrheal activity on castor oil induced diarrhea at all tested doses as compared with the negative control (Table 1).

**Table 1:** Effect of the fractions of *Croton macrostachyus* leaves on castor oil induced diarrheal model in mice

Group	Onset time of diarrhea (min)	Total # of wet feces in 4 h	Total # of feces	Total weight of feces(gm)	% inhibition of defecation
Control	76 ± 16.71	4.5 ± 0.72	4.67 ± 0.80	0.82 ± 0.19	----
Loperamide 3 mg/kg	167.83 ± 25.62^a^*	0.833 ± 0.31^a^***	1.83 ± 0.65^a^*	0.17 ± 0.044^a^*	81.49
CF300mg/kg	169.83 ± 22.78^a^*	1.33 ± 0.42^a^***	1.83 ± 0.65^a^*	0.29 ± 0.14^a^*	70.37
CF400mg/kg	217.50 ± 22.50^a^***	0.33 ± 0.33^a^***	0.83 ± 0.31^a^**	0.14 ± 0.13^a^*	92.59
CF500mg/kg	238.33 ± 1.67^a^***	0.17 ± 0.17^a^***	0.167 ± 0.166^a^***	0.10 ± 0.10^a^*	96.296
Control	82.5 ± 19.96	4.67 ± 0.49	5.33 ± 0.42	0.83 ± 0.22	----
Loperamide 3 mg/kg	167.83 ± 25.62^a^*	0.833 ± 0.31^a^***	1.83 ± 0.65^a^*	0.17 ± 0.044^a^*	82.16
MF300mg/kg	143.5 ± 25.54	2.5 ± 0.56	3.83 ± 0.48	0.37 ± 0.15	46.47
MF400mg/kg	186.33 ± 22.19^a^*	1.17 ± 0.48^a^**	2.50 ± 0.85^a^*	0.114 ± 0.05^a^*	74.95
MF500mg/kg	191.33 ± 30.62^a^*	0.83 ± 0.48^a^***	1.17 ± 0.60^a^*	0.11 ± 0.06^a^*	82.23
Control	42.50 ± 4.16	7.00 ± 0.89	6.67 ± 0.80	0.72 ± 0.13	--------
Loperamide 3 mg/kg	120.50 ± 28.07^a^*	1.50 ± 0.22^a^**^c^**^d^**^e^*^f^*	2.00 ± 0.37^a^*^c^*^d^*^e^*	0.15 ± 0.07^a^*^c^**^d^**^e^**	78.57
AF300mg/kg	54.17 ± 8.60^h^**^I^**	6.83 ± 0.83^b^**^g^***^h^***^I^***	8.17 ± 1.66^b^**^g^***^h^***^I^***	0.71 ± 0.096^b^**^g^***^h^***^I^***	2.43
AF400mg/kg	55.00 ± 8.79 ^h^**^I^**	6.67 ± 1.05^b^**^g^***^h^***^I^***	7.33 ± 0.99^b^*^g^***^h^***^I^***	0.69 ± 0.21^b^**^g^***^h^***^I^***	4.71
AF500mg/kg	60.67 ± 2.58 ^h^**^I^**	6.50 ± 1.09^b^*^g^***^h^***^I^***	7.17 ± 1.11^b^*^g^***^h^***^I^***	0.68 ± 0.06^b^**^g^***^h^***^I^***	7.14
AF1000mg/kg	82.17 ± 28.13 ^h^**^I^**	6.17 ± 1.30^b^*^g^***^h^***^I^***	6.33 ± 1.17^b^*^g^***^h^***^I^***	0.56 ± 0.11	11.86

Values are expressed as Mean ± S.E.M (*n* = 6), analysis was performed with One-Way ANOVA followed by Tukey test,^a^ compared to control,^b^ to standard drug,^c^ to 300 mg/kg,^d^ to 400 mg/kg,^e^ to 500 mg/kg,^f^ to 1000 mg/kg,^g^ to CF300mg/kg,^h^ to CF400mg/kg and^I^ to CF500mg/kg; *P <0.05, **P <0.01, ***P <0.001; *CF* chloroform fraction, *MF* methanol fraction, *AF* aqueous fraction. Negative controls were treated with the vehicle used for reconstitution (2 % v/v Tween 80 for the chloroform and methanol fractions or distilled water for the aqueous fraction) orally

### Effects on castor oil- induced enteropooling in mice

In gastrointestinal enteropooling test, the chloroform and methanol fractions of *C. macrostachyus* leaves reduced the volume of intestinal fluid and weight of the intestinal contents significantly in a dose-dependent manner. Maximum percentage inhibition of the volume of intestinal contents was observed at 500 mg/kg, being 76.1 % (*p* <0.01) and 75.3 % (*P* <0.01) for chloroform and methanol fractions, respectively. Similarly, the uppermost reduction for the weight of intestinal contents was observed at 500 mg/kg for both chloroform and methanol fractions. However, there was no statistically significant difference in the volume of intestinal fluid and weight of intestinal contents when all doses of the chloroform and methanol fractions were compared with Loperamide. On the contrary, the aqueous fraction did not show significant inhibitory effect on the volume and weight of intestinal fluid as compared with the negative control (Table 2).

**Table 2:** Effect of the fractions of *Croton macrostachyus* leaves on castor oil induced enteropooling in mice

Dose administered	Volume of intestinal content (ml)	% of inhibition	Mean weight of intestinal content(gm)	% of inhibition
Control	0.67 ± 0.21	-------	0.78 ± 0.16	-------
Loperamide 3 mg/kg	0.12 ± 0.08^a^**	82.1	0.21 ± 0.08^a^**	73.08
CF300mg/kg	0.18 ± 0.054^a^*	73.13	0.27 ± 0.09^a^**	65.38
CF400mg/kg	0.17 ± 0.03^a^*	74.6	0.269 ± 0.06^a^**	65.51
CF500mg/kg	0.16 ± 0.04^a^**	76.12	0.248 ± 0.04^a^**	68.21
Control	0.77 ± 0.17	-------	0.83 ± 0.2	-------
Loperamide 3 mg/kg	0.12 ± 0.08^a^***	84.41	0.21 ± 0.078^a^**	74.70
MF300mg/kg	0.30 ± 0.05^a^*	61.04	0.36 ± 0.11^a^*	56.63
MF400mg/kg	0.20 ± 0.04^a^**	74.03	0.31 ± 0.04^a^*	62.65
MF500mg/kg	0.19 ± 0.02^a^**	75.32	0.29 ± 0.03^a^*	65.06
Control	0.47 ± 0.06	-------	0.45 ± 0.07	-------
Loperamide 3 mg/kg	0.083 ± 0.01^a^**^c^*^d^*^e^*^f^*	82.34	0.08 ± 0.01^a^*^c^*^d^*^e^*^f^*	82
AF300mg/kg	0.43 ± 0.098^b^*	8.5	0.44 ± 0.08^b^*	2.22
AF400mg/kg	0.38 ± 0.07^b^*	19.12	0.43 ± 0.12^b^*	4.44
AF500mg/kg	0.362 ± 0.09^b^*	22.98	0.422 ± 0.09^b^*	6.22
AF1000mg/kg	0.35 ± 0.06^b^*	25.53	0.41 ± 0.05^b^*	8.89

Values are expressed as Mean ± S.E.M (*n* = 6), analysis was performed with One-Way ANOVA followed by Tukey test;^a^ compared to control,^b^ to standard drug,^c^ to 300 mg/kg,^d^ to 400 mg/kg,^e^ to 500 mg/kg,^f^ to 1000 mg/kg,^g^ to CF300 mg/kg,^h^ to *CF* 400 mg/kg and^I^ to CF500 mg/kg; *P <0.05, **P <0.01, ***P <0.001; *CF* chloroform fraction, *MF* methanol fraction, *AF* aqueous fraction. Negative controls were treated with the vehicle used for reconstitution (2 % v/v Tween 80 for the chloroform and methanol fractions or distilled water for the aqueous fraction) orally

### Effects on castor oil- induced intestinal transit in mice

The chloroform fraction significantly inhibited gastrointestinal transit time of charcoal meal at 300 (25.5 %, *p* <0.05), 400 (43.4 %, *p* <0.01), and 500 (52.4 %, *p* <0.01) mg/kg in comparison to the control. On the other hand, the methanol fraction of *C. macrostachyus* leaves had statistically significant inhibitory effect (56.1 %, *p* <0.001) on gastrointestinal transit time of charcoal meal only at the dose of 500 mg/kg. Interestingly, compared to the control, the aqueous fraction showed significant inhibition of gastrointestinal transit (44.3 %, *p* <0.05) of charcoal meal at the dose of 1000 mg/kg (Table 3).

**Table 3:** Effect of the fractions of *Croton macrostachyus* leaves on castor oil induced intestinal transit in mice

Dose administered	Mean length of small intestine (cm)	Mean Distance traveled by the charcoal meal (cm)	Peristalsis index (%)	% of inhibition
Control	58.75 ± 2.48	55.32 ± 2.78	94.04 ± 1.74	------
Atropine sulphate 5 mg/kg (i.p)	60.27 ± 0.46	18.92 ± 2.59^a^***^c^**	31.33 ± 4.22^a^***^c^**	65.78
CF300mg/kg	57.57 ± 2.38	41.22 ± 0.83^a^*^b^**	72.25 ± 3.42^a^*^b^**	25.49
CF400mg/kg	58.12 ± 2.18	31.33 ± 4.89^a^**	54.56 ± 8.71^a^**	43.36
CF500mg/kg	61.03 ± 0.945	29.00 ± 7.09^a^**	47.37 ± 11.15^a^**	52.42
Control	60.05 ± 2.06	56.88 ± 2.73	94.55 ± 1.78	-------
Atropine sulphate 5 mg/kg (i.p.)	63.1 ± 1.32	24.13 ± 4.24^a^***^c^*	38.71 ± 7.45^a^***^c^*	57.58
MF300mg/kg	61.25 ± 1.52	43.25 ± 5.45^b^*	70.12 ± 8.38^b^*^e^*	23.96
MF400mg/kg	66.28 ± 2.49	42.87 ± 5.89	64.59 ± 8.36	24.63
MF500mg/kg	63.07 ± 1.88	25.00 ± 3.39^a^***	39.78 ± 5.66^a^***^c^*	56.05
Control	62.68 ± 3.54	50.65 ± 3.88	80.81 ± 4.02	-------
Atropine sulphate 5 mg/kg(i.p.)	61.05 ± 2.86	18.72 ± 3.99^a^*^c^*^d^*	30.66 ± 6.26^a^*^c^*^d^*	63.04
AF300mg/kg	63.55 ± 1.47	50.08 ± 2.65^b^*	78.8 ± 6.04^b^*^f^**	1.12
AF400mg/kg	63.45 ± 1.54	49.25 ± 5.76^b^*^f^*	77.62 ± 8.54^b^*^f^**	2.76
AF500mg/kg	61.98 ± 3.69	38.23 ± 7.74^f^*	61.68 ± 11.67	24.5
AF1000mg/kg	68.00 ± 1.53	27.45 ± 2.21^a^*^c^*^d^*	40.53 ± 3.55^a^*^c^**^d^**	44.26

Values are expressed as Mean ± S.E.M (*n* = 6), analysis was performed with One-Way ANOVA followed by Tukey test;^a^ compared to control,^b^ to standard drug,^c^ to 300 mg/kg,^d^ to 400 mg/kg,^e^ to 500 mg/kg,^f^ to 1000 mg/kg,^g^ to CF300 mg/kg,^h^ toCF 400 mg/kg and^I^ to CF500 mg/kg; *P <0.05, **P <0.01, ***P <0.001; *CF* chloroform fraction, *MF* methanol fraction, *AF* aqueous fraction. Negative controls were treated with the vehicle used for reconstitution (2 % v/v Tween 80 for the chloroform and methanol fractions or distilled water for the aqueous fraction) orally

### In vivo anti-diarrheal index

**Fig. 1:**
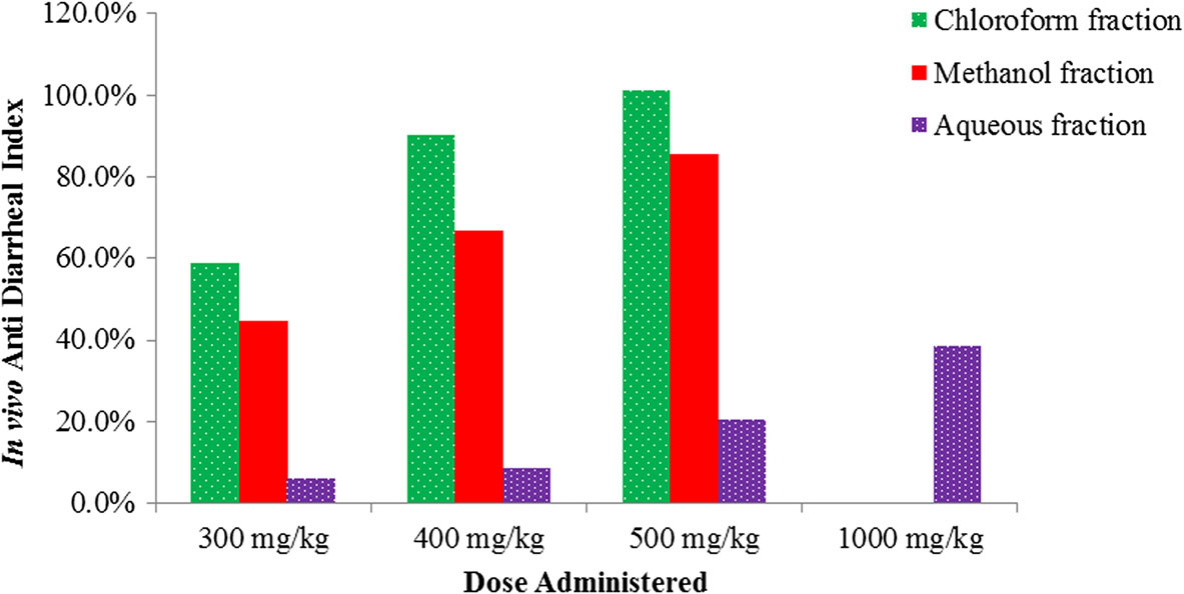
In vivo anti-diarrheal index of the fractions of *Croton macrostachyus* leaves

There was a dose dependent increase in vivo anti-diarrheal index in chloroform and methanol fractions of *C. macrostachyus* leaves. The highest anti-diarrheal index was observed at the maximum dose of each fraction. However, amongst all solvent fractions of *C. macrostachyus* leaves, the chloroform fraction showed the highest anti-diarrheal index at 500 mg/kg (Fig. 1).

### Preliminary phytochemical screening

Phytochemical screening of solvent fractions C*. macrostachyus* leaves revealed that the presence of alkaloids, steroids and Terpenoids in the chloroform fraction. On the other hand, the methanol fraction possessed alkaloids, Saponins, tannins, flavonoids, and cardiac glycosides whereas the aqueous fraction was found to contain Saponins, Tannins and alkaloids (Table 4).

**Table 4:** Preliminary phytochemical screening of solvent fractions of *Croton macrostachyus* leaves

Secondary metabolites	Chloroform fraction	Methanol fractions	Aqueous fraction
Alkaloids	+	+	+
Tannins	−	+	+
Saponins	−	+	+
Terpenoids	+	−	−
Steroids	+	−	−
Flavonoids	−	+	−
Anthraquinones	−	−	−
Cardiac Glycosides	−	+	−

+ present, − absent

## Discussion

The use of castor oil as diarrhea inducer is well documented [25, 35, 36]. Its active metabolite, ricinoleic acid, is responsible for the diarrhea inducing properties and is liberated by the action of lipases in the upper part of the small intestine [37, 38].

Ricinoleic acid produces local irritation and inflammation of the intestinal mucosa, causing the release of prostaglandins that eventually increase gastrointestinal motility, net secretion of water and electrolytes [39, 40]. This effect could occur due to the capability of ricinoleic acid to activate the G protein-coupled prostanoid receptor (EP3) on the smooth muscle cell of the intestine [41]. In addition, it forms ricinoleate salts with sodium and potassium in the lumen of the intestine and these salts inhibit sodium-potassium ATPase and increase permeability of the intestinal epithelium, which in turn produces a cytotoxic effect on intestinal absorptive cells [37].

In the castor oil induced diarrheal model, the chloroform (at all tested doses) and methanol fractions (at 400 and 500 mg/kg) significantly delayed the time of diarrheal onset, and decreased the frequency of defecation and weight of feces. Moreover, the chloroform fraction showed comparable effect with that of the crude extract in this model [42]. The findings from this study are in line with other studies which show that the chloroform extract of different plants reduced the stool frequency in a dose-dependent manner [43–45]. In contrast, the aqueous fraction was devoid of any activity at all tested doses. This could possibly suggest the probable localization of the active ingredients in these two fractions.

It is postulated that Non-steroidal anti-inflammatory drugs (NSAIDs) inhibit castor oil -induced prostaglandin production, thereby preventing diarrhoea [46]. Similarly, the aqueous and methylene chloride/methanol extracts from the stem bark of *C. macrostachyus* has been shown to have analgesic and anti-inflammatory activities [20]. It is therefore plausible that the antidiarrheal effect of the active fractions could be attributed to inhibition of castor oil-induced prostaglandin synthesis.

Terpenoids such as abietic acid and steroids like phytosterols have been shown to inhibit production of prostaglandin E2 [47, 48], which are known to play a crucial role in the stimulation of intestinal secretions [49]. Thus, the significant antidiarrheal activity observed in the chloroform fraction could probably be attributed to the presence of these secondary metabolites in the fraction.

Although the chloroform fraction exhibited maximum effect, there was no significant difference in antidiarrheal effects between the chloroform and methanol fractions, indicating that both fractions are active in this model. The present results are concordant with other studies, where the chloroform and methanol fractions displayed comparable inhibition of castor oil induced diarrhea [26, 32].

Despite there appears to be a differential distribution of active constituents in the two fractions, the comparable activity of the two fractions suggests that different secondary metabolites are endowed with antidiarrheal properties.

In the castor oil induced enteropooling test, treatment of mice with different doses of the chloroform and methanol fractions produced a significant decline in the intestinal fluid accumulation. On the contrary, the aqueous fraction did not significantly inhibit castor oil induced intestinal fluid accumulation in all the tested doses. In comparison to the crude extract, [42] both chloroform and methanol fraction demonstrated higher effect in this model.

The active metabolite of castor oil (ricinoleic acid) might activate the nitric oxide pathway and induce nitric oxide (NO) dependent gut secretion [50, 51]. A growing body of evidence indicates that phytochemical constituents such as terpenoids [52] and flavonoids [53–55] are implicated in attenuation of NO synthesis. Thus, in contrast to the aqueous fraction, the pronounced inhibition of castor oil induced intestinal fluid accumulation (enteropooling) and the weight of the intestinal content could possibly be linked to the presence of terpenoids (chloroform fraction) and flavonoids (methanol fraction) that increase the reabsorption of electrolytes and water by hindering castor oil mediated NO synthesis. The fact that intestinal fluid accumulation and Na+ secretion induced by castor oil is attenuated by pretreatment of rats with NO synthesis inhibitors [51] reinforces the notion that the anti-enteropooling effect of both fractions could probably be by interfering with the NO pathway.

Alkaloids, which are detected in all fractions, also demonstrated inhibitory effect on NO synthesis [56]. Nevertheless, due to the successive extraction method used in this study, most of the alkaloids could be extracted by the chloroform and methanol, and trace amounts might have remained in the aqueous fraction. Consequently, the phytochemical constituents that could inhibit castor oil induced fluid secretion were either absent or present in undetectable amount in the aqueous fraction, explaining why this fraction had lower antidiarrheal effects.

The antidiarrheal effect of flavonoids has been ascribed to their ability to inhibit intestinal motility and hydro-electrolytic secretion [57–59]. Flavonoids are also able to inhibit the intestinal secretory response induced by prostaglandins E2 [60, 61]. Moreover, the enteric nervous system stimulates intestinal secretion through neurotransmitters such as acetylcholine and vasoactive intestinal peptide. On the other hand, intestinal absorption can be stimulated with alpha two adrenergic agents, enkephalins, and somatostatin [49, 62]. Secondary metabolites such as flavonoids from plant sources could stimulate alpha two adrenergic receptors in the absorptive cells of the gastrointestinal tract [57]. Hence, in contrast to the aqueous fraction, the significant anti-secretory activity of the methanol fraction could probably be related to the existence of flavonoids that in turn stimulate alpha two adrenergic receptors in the enterocytes and promote fluid and electrolyte absorption. However, the chloroform and methanol fractions showed comparable effect in this model despite the better antidiarrheal effect observed in the chloroform fraction. This could perhaps be due to the collective interference of terpenoids and steroids on prostaglandin E2 induced gut secretion [47, 48].

Evaluation of intestinal transit, demonstrated a significant reduction in the intestinal propulsive movement of charcoal meal in the chloroform fraction in comparison to the negative control at all the test doses (300,400 and 500 mg/kg body weight). Besides, this fraction showed greater effect in this model as compared to the crude extract in the previous study [42]. This is comparable to other studies, in which the chloroform extract significantly inhibited the distance travelled by charcoal meal [26]. Interestingly, both the methanol fraction (56.1 %, *p* <0.001) as well as the aqueous fraction (44.3 %, *p* <0.05) produced substantial inhibition of the peristaltic movement of charcoal meal at the higher dose employed in the present study (500 and 1000 mg/kg, respectively). This finding suggests that there is a difference in the potency (chloroform>>methanol>>aqueous fraction) of phytochemical constituents that mediate castor oil induced gastrointestinal motility. The potent action of the chloroform fraction could be ascribed to the synergistic effects of terpenoids and alkaloids to prolong the time for absorption of water and electrolytes through hampering peristaltic movement of the intestine. Indeed, alkaloids [63] and terpenoids [63, 64] have been demonstrated to have inhibitory effect on gastrointestinal motility. Although the phytochemical constituents responsible for the antidiarrheal effect are yet to be identified, the amount of phytochemical constituents that are responsible for impeding gastrointestinal motility such as tannins [65, 66] and alkaloids [63] appear to increase with dose. This could possibly the reason why significant anti-motility effect was observed at the higher dose of the aqueous fraction. However, in this fraction, significant antidiarrheal effect was not observed in other models with increasing in dose. This might be due to the lack of secondary metabolites such as terpenoids [48], steroids [47] and flavonoids [60, 61] that could inhibit prostaglandin E2 induced fluid secretion in the intestine.

Unlike the castor oil induced and enteropooling diarrheal model, maximum effect was observed with the methanol fraction (56.1 %) rather than the chloroform fraction (52.4 %) in charcoal meal test. This could perhaps be linked to the presence of synergistic inhibitory effect of tannins [38, 65, 66] and flavonoids [57–59] on castor oil induced gastrointestinal motility.

Plants that have tannins in their composition can precipitate proteins of the enterocytes, reducing the peristaltic movements and intestinal secretions [38, 65, 66]. The layers formed by the precipitate of proteins on the mucosal surface of the enterocytes also inhibit the development of microorganisms, thus explaining the antiseptic action of tannins [65]. Based on this fact, the presence of tannins in the methanol fraction might provide a clue to further investigate its role for infectious diarrhea, as the present study did not address this issue.

The anti-diarrheal index (ADI) is a measure of the combined effects of the different parameters of diarrhea such as purging frequency, onset of diarrheal stools and intestinal motility [31]. The higher the ADI value the more effective an extract is at curing diarrhea [67, 68]. The findings revealed that ADI increased in a dose dependent manner for all the fractions suggesting the dose dependency of this parameter. The chloroform fraction showed the highest ADI value as compared to the other fractions, reinforcing the notion that this fraction is endowed with the best anti-diarrheal activity among all the solvent fractions. Conversely, the aqueous fraction, which had little or no antidiarrheal activity in most of the models, exhibited the lowest ADI, pointing to the fact that ADI is a useful parameter in ranking antidiarrheal agents.

## Conclusion

The present study revealed that the chloroform and methanol fractions of *C. macrostachyus* leaves possessed significant anti-diarrheal activity. Nevertheless, the aqueous fraction showed only significant anti-motility effect at the higher dose (1000 mg/kg) employed in the study. The antidiarrheal activities of these fractions could be attributed to the presence of bioactive agents, which are either non-polar or semi polar, including, among others, tannins, alkaloids, saponins, flavonoids, steroids, and terpenoids that act individually or collectively. Hence, further studies that aim to isolate the active principle (s) or elucidating the possible mechanism of action should use either fraction.

## Abbreviations

ADI: Anti-diarrheal index
NO: Nitric oxide
NSAIDs: Non- steroidal anti-inflammatory drugs
